# Editorial: Machine Learning Techniques on Gene Function Prediction

**DOI:** 10.3389/fgene.2019.00938

**Published:** 2019-10-04

**Authors:** Quan Zou, Arun Kumar Sangaiah, Dariusz Mrozek

**Affiliations:** ^1^Institute of Fundamental and Frontier Sciences, University of Electronic Science and Technology of China, Chengdu, China; ^2^School of Computing Science and Engineering, VIT University, Vellore, India; ^3^Institute of Informatics, Silesian University of Technology, Gliwice, Poland

**Keywords:** machine leaming, gene function prediction, deep learning, ensemble learning, bioinformatics

Gene function, including that of coding and noncoding genes, can be difficult to identify in molecular wet laboratories. Therefore, computational methods, often including machine learning, can be a useful tool to guide and predict function. Although machine learning has been considered as a “black box” in the past, it can be more accurate than simple statistical testing methods. In recent years, deep learning and big data machine learning techniques have developed rapidly and achieved an amazing level of performance in many areas, including image classification and speech recognition. This Research Topic explores the potential for machine learning applied to gene function prediction.

We are pleased to see that authors brought the latest machine learning techniques on gene function prediction. Submissions came from an open call for paper, and they were accepted for publication with the assistance of professional referees. Forty-six papers are finally selected from a total of 72 submissions after rigorous reviews. They were presented from different countries and regions, including China, USA, Poland, Taiwan, Korea, Saudi Arabia, India, and so on. According to the topics, we categorize three subtopics for our special issue.

The first part of this special issue discusses the gene and disease relationship. Six papers included in this part are focused on general diseases. These papers propose novel methods to predict disease and gene/miRNA/long noncoding RNA (lncRNA) associations. Su et al. proposed a novel method called GPSim to effectively deduce the semantic similarity of diseases. Yu et al. constructed a weighted four-layer disease–disease similarity network to characterize the associations at different levels between diseases. Three papers paid attention to miRNA and disease relationship. Qu et al. proposed a novel method to predict miRNA–disease associations based on Locality-constrained Linear Coding. Zhao et al. proposed a novel computational model of SNMFMDA (Symmetric Nonnegative Matrix Factorization for MiRNA-Disease Association prediction) to reveal the relation of miRNA–disease pairs. He et al. proposed an NRLMFMDA (neighborhood regularized logistic matrix factorization method for miRNA–disease association prediction) by integrating miRNA functional similarity, disease semantic similarity, Gaussian interaction profile kernel similarity, and experimental validation of disease–miRNA association. Besides miRNA, there is still a paper on lncRNA–disease relationship prediction. A dual-convolutional neural networks with attention mechanism–based method are presented for predicting the candidate disease lncRNAs (Xuan et al.).

There are seven papers on cancer and oncogenes. Two papers paid attention to cancer subtypes. Liu et al. classified muscle-invasive bladder cancer into two conservative subtypes using miRNA, mRNA, and lncRNA expression data; investigated subtype-related biological pathways; and evaluated the subtype classification performance using machine learning methods. Jiang et al. employed spectral clustering and a novel kernel to predict cancer subtypes. Two papers are focused on breast cancer. Abou Tabl et al. present a hierarchical machine learning system that predicts the 5-year survivability of the patients who went through specific therapy. Li et al. employed machine learning methods to select 54 novel breast cancer oncogenes and proved their findings with GO and KEGG. Three papers researched on other kinds of cancer. Liu et al. found lncRNA LINC00941 as a potential biomarker of gastric cancer. Gao et al. proposed an ensemble strategy to predict prognosis in ovarian cancer. Guo et al. developed rigorous bioinformatics and statistical procedures to identify tumor-infiltrating bacteria associated with colorectal cancer.

Two papers focused on type 2 diabetes and four papers paid attention to other diseases. Zhuang et al. employed a two-sample Mendelian randomization method to analyze the causal relationships between interleukin 18 (IL-18) plasma levels and type 2 diabetes using IL-18–related SNPs (Single Nucleotide Polymorphism) as genetic instrumental variables. Sun et al. establish a multilevel comparative framework across three insulin target tissues (white adipose, skeletal muscle, and liver) to provide a better understanding of type 2 diabetes. Zhong et al. identified potential prognostic genes for neuroblastoma. Wang et al. predicted chronic kidney disease susceptibility gene PRKAG2 by comprehensive bioinformatics analysis. Lu et al. employed the Laplacian heat diffusion algorithm to infer novel genes with functions related to uveitis. Li et al. analyzed the blood gene expression signature for osteoarthritis with advanced feature selection methods.

The second part focused on gene structure and function prediction. Four papers were involved in gene elements, and two papers researched RNA structure. Oubounyt et al. employed deep learning techniques to predict gene promoter regions. Dao et al. gave a review for detecting DNA replication origins in eukaryotic genomics with machine learning methods. Exons skipping is an important issue in gene structure research. Chen, Feng et al. and Chen, Song et al. analyzed the relationship between histone modifications and exons skipping. Two papers performed researches on RNA secondary structure prediction, which is a classical problem in computational biology. Wang et al. and Zhang et al. employed deep learning to predict RNA secondary structure, especially on pseudoknots.

Besides gene structure prediction, four papers focused on the gene function prediction, and five papers paid attention to gene identification. Due to the GO- and KEGG-rich knowledge for gene function, researchers would like to pay attention to noncoding RNA function prediction. Zhang et al. predicted noncoding RNA function with deep learning network. Zhao and Ma employed Multiple Partial Regularized Nonnegative Matrix Factorization for Predicting Ontological Functions of lncRNAs. Deng et al. proposed an integrated model to infer the gene ontology functions of miRNAs The work was supported by the National Key R&D Program of China (2018YFC0910405), the Natural Science Foundation of China (No. 61771331). by integrating multiple data sources. Zou et al. predicted enzyme function with hierarchical multilabel deep learning.

There are also five papers on gene identification, expression pattern prediction, and sites modification. They are all involved with machine learning techniques. Han et al. predicted ion channels genes and their types. Chen et al. paid attention to MADS-box gene classification and clustering. Liu et al. predicted gene expression patterns with a generalized linear regression model. Fu et al. identified microRNA genes with sequence and structure information. Qiang et al. predicted RNA N6-methyladenosine sites with machine learning and sequence features.

Other researches were categorized as the third part of our special issue. There are 12 papers in total in this part. Two papers are focused on drugs. Zhu et al. predicted drug–gene interactions with Metapath2vec. Xuan et al. resolve this problem with the latest machine learning technique gradient boosting decision tree. Four papers researched lncRNA–protein interaction prediction. Xie et al. predicted this problem with improved bipartite network recommender algorithm. Zhan et al. combined sequence and evolutionary information on this problem. Zhao et al. employed random walk and neighborhood regularized logistic matrix factorization approach. Dai et al. paid attention to complex features for ncRNA–protein interaction prediction. Three papers are focused on plant researches. Qu et al. found effective sequence features for classifying plant pentatricopeptide repeat proteins. Jiang et al. identified rice yield-related candidate genes by walking on the functional network. Zhang et al. mined *Magnaporthe oryzae* sRNAs with potential transboundary regulation of rice genes associated with growth and defense through expression profile analysis of the pathogen-infected rice. Three papers paid attention to RNA-seq data analysis. McDermaid et al. proposed a new machine learning–based framework for mapping uncertainty analysis in RNA-seq read alignment and gene expression estimation. Wang et al. gave a systems analysis of the relationships between anemia and ischemic stroke rehabilitation based on RNA-seq data. Niu et al. developed rSeqTU, which is a machine learning–based R package for predicting bacterial transcription units from RNA-seq data.

To conclude, papers in this special issue cover several emerging topics of advanced learning techniques and applications for bioinformatics. We highly hope this special issue can attract concentrated attention in the related fields. We thank the reviewers for their efforts to guarantee the high quality of this special issue. Finally, we thank all the authors who have contributed to this special issue.

## Author Contributions

ZQ wrote the manuscript draft. DM helped to revise the text. AKS gave some helpful suggestions. 

## Funding

The work was supported by the National Key R&D Program of China (2018YFC0910405), the Natural Science Foundation of China (No. 61771331, No. 61922020), Statutory Research funds of Institute of Informatics, Silesian University of Technology, Gliwice, Poland (BK/204/RAU2/2019), and the professorship grant of the Rector of the Silesian University of Technology (02/020/RGPL9/0184).

## Conflict of Interest

The authors declare that the research was conducted in the absence of any commercial or financial relationships that could be construed as a potential conflict of interest.

